# Modeling the effect of blunt impact on mitochondrial function in cartilage: implications for development of osteoarthritis

**DOI:** 10.7717/peerj.3468

**Published:** 2017-07-17

**Authors:** Georgi I. Kapitanov, Bruce P. Ayati, James A. Martin

**Affiliations:** 1 Department of Mathematics, University of Iowa, Iowa City, IA, United States of America; 2 Program in Applied Mathematical & Computational Sciences, University of Iowa, Iowa City, IA, United States of America; 3 Department of Orthopedics & Rehabilitation, University of Iowa, Iowa City, IA, United States of America; 4 Department of Biomedical Engineering, University of Iowa, Iowa City, IA, United States of America

**Keywords:** Osteoarthritis, Modeling & simulation, Oxidative stress, Mitochondria, Articular cartilage

## Abstract

**Objective:**

Osteoarthritis (OA) is a disease characterized by degeneration of joint cartilage. It is associated with pain and disability and is the result of either age and activity related joint wear or an injury. Non-invasive treatment options are scarce and prevention and early intervention methods are practically non-existent. The modeling effort presented in this article is constructed based on an emerging biological hypothesis—post-impact oxidative stress leads to cartilage cell apoptosis and hence the degeneration observed with the disease. The objective is to quantitatively describe the loss of cell viability and function in cartilage after an injurious impact and identify the key parameters and variables that contribute to this phenomenon.

**Methods:**

We constructed a system of differential equations that tracks cell viability, mitochondrial function, and concentrations of reactive oxygen species (ROS), adenosine triphosphate (ATP), and glycosaminoglycans (GAG). The system was solved using MATLAB and the equations’ parameters were fit to existing data using a particle swarm algorithm.

**Results:**

The model fits well the available data for cell viability, ATP production, and GAG content. Local sensitivity analysis shows that the initial amount of ROS is the most important parameter.

**Discussion:**

The model we constructed is a viable method for producing in silico studies and with a few modifications, and data calibration and validation, may be a powerful predictive tool in the search for a non-invasive treatment for post-traumatic osteoarthritis.

## Introduction

Osteoarthritis (OA) is a degenerative disease characterized by thinning of the joint cartilage and is associated with disability and pain. Chronic elevated contact stresses and strains between joints have been implicated in the pathogenesis of OA and are often age-related (wear and tear) or due to continuous injurious loading (e.g., running on hard surfaces) (Buckwalter, Mankin & Grodzinsky, 2005). Osteoarthritis can also occur after a single forceful impact injury, in which case it is referred to as post-traumatic osteoarthritis (PTOA). We hypothesize that the biochemical processes associated with PTOA are the same as those that lead to OA, only occurring on a different time scale: While age-related OA can take decades to occur, PTOA can develop in a matter of months (Anderson et al., 2011; Buckwalter & Felson, 2015). At the same time, cartilage biomechanics dictates that some level of stress is important for normal development and stability of the cartilage surface - inactivity can also lead to OA development (Martin & Buckwalter, 2012; Tomiyama et al., 2007).

Current treatment options for OA are not ideal. When the cartilage is severely worn out, usually in elderly patients, a whole joint replacement is advised. This strategy has been quite successful, but cannot and should not be implemented in less severe cases. For those, a surgical intervention requires a surgeon to manually adjust the joint as to mitigate the contact stresses and direct them to areas of the cartilage that is less damaged. Currently, this is largely an approach based on the surgeon’s experience and intuition as has resulted in limited efficacy (Anderson et al., 2011). Another approach is painkillers or anti-inflammatory compounds. While they may help the pain, they have shown to do little in the way of preventing or even slowing down the disease (Kongtharvonskul et al., 2016). Therefore, there is a need for non-invasive strategies for preventing or treating OA, and a goal of this article is to provide a model that assists the processes of understanding and quantifying the underlying biological mechanisms that lead to OA development.

There are generally two hypotheses as to the biochemical source of degeneration in OA. One is the role of joint inflammation and particularly the disruption of the balance between pro- and anti- inflammatory cytokines in the joint (Scanzello, 2017). Our group has been leading the modeling efforts in this area with several publications (Graham et al., 2012; Wang et al., 2014; Wang et al., 2015; Ayati et al., 2017; Kapitanov et al., 2016). In Graham et al. (2012), the authors laid out the theoretical work for a temporal-spatial model for the degeneration of a cartilage explant (a cartilage cylinder containing subchondral bone) after a blunt impact. They were able to qualitatively capture the radial degeneration of the cartilage cylinder after the impact. Better parametrization and data validation was done in Wang et al. (2015), and adding an external map of the strains that result from the impact and their relationship to cell death was added in Kapitanov et al. (2016). The problem with cyclic loading onto an explant (imitating a continuous activity, like running) was modeled in Wang et al. (2014) and Ayati et al. (2017). The latter also gives an overview on how combining modeling and simulation research related to the mechanical properties of cartilage and the joint stress map after loading, and modeling and simulation of the underlying cascade of biochemical reactions that can lead to OA, can eventually be translated to patient outcomes and treatment strategies, using mathematics as a conduit in this complicated process.

Recent research efforts by the University of Iowa Department of Orthopedics and Rehabilitation have identified a different chondrocyte-centered hypothesis for the development of OA: oxidative stress and particularly the disruption of mitochondrial function as a result of joint overload (Coleman, Buckwalter & Martin, 2015; Goetz et al., 2017; Coleman et al., 2016). This hypothesis can explain the unsuccessful implementation of anti-inflammatories in treating OA, and reveals other possibilities for non-invasive interventions. The present work is the first attempt known to us to model this aspect of the underlying biochemistry after blunt impact and can be useful as a stepping stone to quantifying the treatment options that will result from the hypothesis. The article is organized as follows: the ‘Materials & Methods’ section presents the laboratory experimental set-up from already published work, the hypotheses, and the resulting mathematical model; the ‘Results’ section presents our results; the ‘Discussion’ section is a discussion.

## Materials & Methods

This section details the experimental set-up we are modeling, the biological hypotheses involved, the resulting model, and the computational work involved in solving and fitting the model to the experimental data.

### Laboratory experiments

Our modeling efforts revolve around laboratory experiments outlined in Martin et al. (2009) and Coleman, Buckwalter & Martin (2015). Briefly, osteochondral explants (pieces of articular cartilage with subchondral bone underneath, harvested from cattle), are secured at the bottom of a drop tower and subjected to high-energy blunt impacts (from a 5 mm diameter brass rod dropped onto the explant from different heights) of different magnitudes, comparable to those estimated to occur in serious joint injuries (7 J/cm^2^ and 14 J/cm^2^). The effects of impacts on cell viability within 72 h post-impact were recorded by putting the explants into media containing calcein acetoxymethylester (which dyes live cells green) and ethidium homodimer-2 (which dyes dead cells red), taking images with confocal microscopy and analyzing the images from six areas of three explants (eighteen images total per time point) (Martin et al., 2009). The viability was recorder as % live cells (green) to total cells (green plus red).

Cartilage is a tissue that comprises of mainly extracellular matrix with a dispersion of chondrocytes throughout. The extracellular matrix is composed of water, collagen, and proteoglycans, in different proportions depending on the cartilage depth (more proteoglycans toward the bottom and less toward the top, which makes it heterogeneous in stiffness—stiffer as one transitions from top to bottom). The effects of 7 J/cm^2^ impacts on proteoglycans was measured by glycosaminoglycan (GAG) assay with dimethyl methylene blue. Proteoglycans contains GAG molecules, so GAG content is an indicator of cartilage stiffness and stability (Coleman, Buckwalter & Martin, 2015). The relative GAG content (GAG at impact versus GAG at nearby undamaged site) was measured at 7 and 14 days post-impact and averaged over six explants. Metabolic activity and energy metabolism, as revealed by adenosine triphosphate (ATP) content, was assessed at three different sites (impact, near impact, remote) at 24 h and 48 h post 7 J/cm^2^ impact. No ATP or GAG data was collected for the 14 J/cm^2^ impact. In this model we only consider the results at the impact site because that is consistent with the viability and GAG data available.

### Biological hypothesis

Cartilage is a hypoxic tissue—while most human cells’ mitochondria use an oxidant-rich process of phosphorylation to produce ATP, chondrocytes’ mitochondria produce ATP through glycolisis, which requires small amounts of oxygen. Therefore, in articular cartilage there is a fine balance between the oxidants needed for normal cellular metabolism and an excess that can cause metabolic damage. High energy impacts to cartilage cause local oxidative chondrocyte death (Martin et al., 2009), as well as a decline in ATP production (Figure 17.1 in Coleman, Buckwalter & Martin, 2015). These effects were found to be related to excessive production of reactive oxygen species (ROS) by the mitochondrial electron transport chain, which causes damage to chondrocyte mitochondria and oxidative stress that inhibits glycolytic activity. The resulting loss of ATP affects many cellular activities, but most notably it diminishes the production of GAGs, which undermines the stability of the cartilage matrix. The subject of the present work is describing these processes mathematically and creating a mechanistic model that qualitatively and quantitatively describes their effects on the progression of PTOA.

### Mathematical model

Based on the biological hypothesis and the available data, we constructed a system of differential equations that describes the interactions between the mechanical impact from the drop tower, the mitochondrial function of the chondrocytes in the impact area, and the resulting concentrations of ROS, ATP, and GAG. The model is unitless to reflect the available data (% of cell viability for example).

We include the following variables:

1. *M*(*t*): proportion of live cells with functional (normal, undamaged) mitochondria in the cartilage explant impact area.
2. *D*(*t*): proportion of live cells with dysfunctional (damaged, abnormal) mitochondria in the cartilage explant impact area. These cells are characterized by their release of double the amount of ROS as cells with functional mitochondria.
3. *R*(*t*): relative concentration of reactive oxygen species (ROS) in the cartilage explant impact area.
4. *E*(*t*): relative concentration of ATP (produced through glycolisis) in the cartilage explant impact area.
5. *U*(*t*): relative concentration of GAG (measure of cartilage stability) in the cartilage explant impact area.

**Figure 1:**
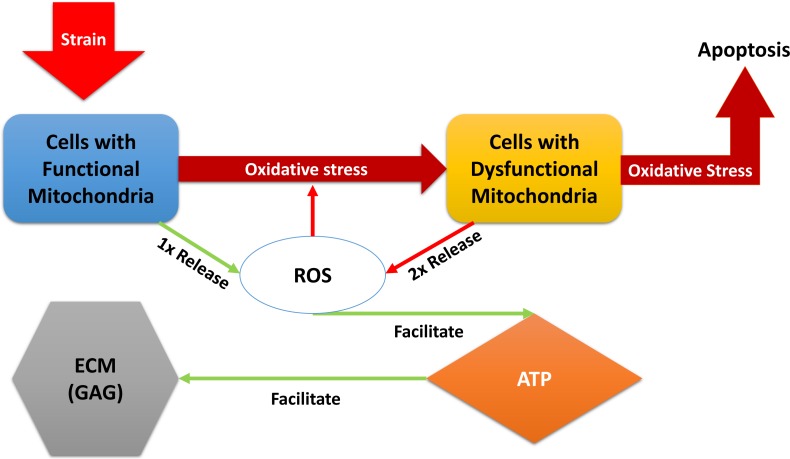
A diagram of the dynamics expressed in Eq. (1). External strain from the blunt impact causes cells with functional mitochondria to transition into cells with dysfunctional mitochondria and cells with dysfunctional mitochondria to go into apoptosis. Cells with dysfunctional mitochondria release twice the amount of reactive oxygen species (ROS) as normal cells, which further affects the oxidative stress. ROS is used in production of ATP, which in turn is utilized for the release of glycosaminoglycans (GAG), which strengthen the ECM.

“Relative” in the descriptions above refers to comparison with normal, undamaged cartilage explant (control in the experiments). A brief description of the models dynamical system of equations below: the blunt impact causes a burst of ROS, which creates an environment of oxidative stress. The stress causes the mitochondria of normal chondrocytes to become dysfunctional, which makes them release additional ROS, and can cause cellular apoptosis. The amount of ROS that is available controls the ATP formation in the cell if the concentration of available oxidants is too little or too high, the ATP production is decreased (Coleman, Buckwalter & Martin, 2015). Decreased ATP production negatively affects GAG production and hence concentration in the impact area. A diagram of the interactions is presented in Fig. 1. We assume no cell proliferation—none was observed during the experiments, and the experiment’s time frame would suggest no significant number of new cells has been added. We consider 1 to be a level of the relevant variable that is considered optimal for cartilage function. This idea translates to assuming that when 100% (or a fraction of 1 of the total number of cells) of the viable cells have functional mitochondria, the released amount of ROS is 1, which translates to an optimal ATP production (assumed to be 1), and overall cartilage integrity (relative GAG concentration of 1). In other words the control case is assumed to be the case where *M*(*t*) = 1, *D*(*t*) = 0, *R*(*t*) = 1, *E*(*t*) = 1, and *U*(*t*) = 1. The control case is trivial, hence not pictured in the figures that accompany the model.

(1) \begin{eqnarray*}\begin{array}{@{}ll@{}} \displaystyle \frac{dM}{dt} =&\displaystyle \underbrace{-{k}_{S}MS(R){}}_{\text{mito damage due to ox. stress}}\\ \displaystyle \frac{dD}{dt} =&\displaystyle \underbrace{{k}_{S}MS(R){}}_{\text{mito damage due to ox. stress}}-\underbrace{{\delta }_{D}DS(R){}}_{\text{apoptosis due to ox. stress}}\\ \displaystyle \frac{dR}{dt} =&\displaystyle \underbrace{{\alpha }_{M}(M+{k}_{D}D){}}_{\text{mito ROS release}}-\underbrace{{\delta }_{R}R{}}_{\text{ROS clearance}}\\ \displaystyle \frac{dE}{dt} =&\displaystyle \underbrace{{f}_{E} \left( \frac{R}{M+D+\epsilon } \right) {}}_{\text{ATP production}}-\underbrace{{\delta }_{E}E{}}_{\text{utilization}}\\ \displaystyle \frac{dU}{dt} =&\displaystyle \underbrace{{k}_{U}U(1- \frac{1+{\lambda }_{U}}{1+{\lambda }_{U}E} U){}}_{\text{GAG through ATP}}. \end{array}\end{eqnarray*}

The function *S*(*R*) represents the effect of oxidative stress on the system. It only triggers when an excessive amount of ROS is present.

(2) \begin{eqnarray*}S(R)& =& \left\{ \begin{array}{@{}l@{}} \displaystyle 0~\text{if}~R\leq 1 \\ \displaystyle {s}_{C}(R-1)^{\alpha }~\text{if}~R\gt 1. \end{array} \right. \end{eqnarray*}

**Figure 2:**
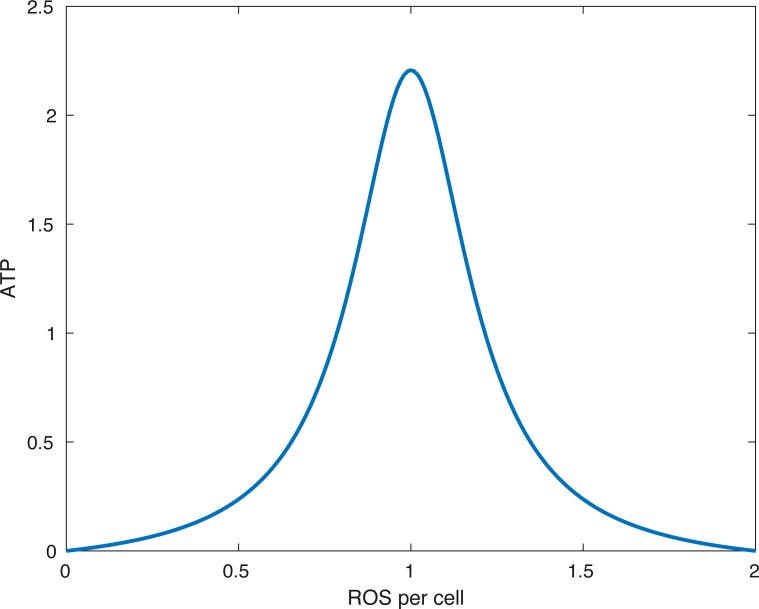
The form of the function *f*_*E*_. The peak (optimal) ATP production occurs when the amount of ROS per cell (*R*∕(*M* + *D*)) is equal to *R*_0_. In the picture above *R*_0_ = 1∕(1.0001).

The constant *s*_*C*_ represents the direct effect of oxidative stress, represented by ROS being above the optimal level of 1, on the mitochondrial function and viability. We choose the constant *α* to be greater than 1. This ensures that *S*(*R*) is smooth at *R* =1 and simplifies the equilibrium analysis.

The function *f*_*E*_(*x*) describes the energy (ATP) production. In our model *x* is the ratio between available ROS and viable cells *R*∕(*M* + *D* + *ϵ*). The parameter *ϵ* > 0 is there to avoid division by 0.

(3) \begin{eqnarray*}{f}_{E}(x)& =& \left\{ \begin{array}{@{}l@{}} \displaystyle 0~\text{if}~x\leq 0~\text{or}~x\geq 2{R}_{0} \\ \displaystyle \frac{{k}_{E}}{(x-{R}_{0})^{2}+{\lambda }_{E}} - \frac{{k}_{E}}{{R}_{0}^{2}+{\lambda }_{E}} ~\text{if}~x\in (0,2{R}_{0}). \end{array} \right. \end{eqnarray*}

What the function *f*_*E*_ describes is that if the relative amount of ROS is below some optimal level *R*_0_ or above it, then the energy production is lower than optimal. Furthermore, no available ROS (*R* = 0) or too much ROS (*R* > 2*R*_0_) shuts down ATP production. This idea is presented in Coleman, Buckwalter & Martin (2015). A plot of the function can be seen in Fig. 2.

### Parameter relationships

We assume that under homeostasis (undamaged cartilage), the values of cell with functional mitochondria, ROS, ATP, and GAG (*M*, *R*, *E*, and *U* respectively) remains 1, while the value for cells with dysfunctional mitochondria, *D*, remains 0. The only reason for changes is disruption of this equilibrium due to an impact. To ensure this equilibrium, the following relationships between parameters were assumed

1. We assumed that in the function *f*_*E*_ that *R*_0_ = 1∕(1 + *ϵ*) so as to produce the maximum amount of ATP when *R* = 1 and *M* + *D* = 1.
2. We considered a level of 100% *M* to be optimal/normal for cartilage. This assumption requires that *α*_*M*_ = *δ*_*R*_, since we seek an equilibrium *R*^∗^ = 1 when *M*^∗^ = 1.
3. With the assumptions above, in order to produce an equilibrium *E*^∗^ = 1 when *R*^∗^ = 1, we assume that ${\delta }_{E}= \frac{{k}_{E}}{{\lambda }_{E}(1+{\lambda }_{E})} $.

### Numerical solutions and data fitting

System Eq. (1) was solved using the MATLAB function ode15s. The parameter values used for generating the results can be seen in Table 1. The data used for parametrization of our model can be seen in Table 2. We note that the data is modified. In the experimental results in Martin et al. (2009), all explants had mean initial viability of 89%, including control. If 89% viability is normal for cartilage, we divided all the data by 89% to get the normal viability to be 100% (or 1 in the simulation calculations). In other words, the viability data was scaled. We assumed that undamaged cartilage only contains cells with functional mitochondria, that ATP, and GAG content are optimal, and the impact increased the initial amount of ROS above 1, depending on the impact’s energy. We fit all parameters, besides *R*(0) for the 14 J/cm^2^ impact, using the 7 J/cm^2^ data in Table 2 (cell viability, ATP, GAG). Then, using the parameters we found, we fit the initial amount of ROS after the 14 J/cm^2^ impact to the cell viability data in that case. We used the MATLAB particle swarm function for fitting the parameters.

### Local sensitivity analysis

Let us denote by *S*_par,var_ the effect of the parameter *par* on the variable *var*. Standard methods of local sensitivity analysis boil down to solving a set of differential equations with respect to *S*_par,var_, namely

\begin{eqnarray*} \frac{d{\vec{S}}_{\text{par}}}{dt} =J\cdot {\vec{S}}_{\text{par}}+F, \end{eqnarray*}

where ${\vec{S}}_{\text{par}}$ is the vector of *S*_par,var_ with respect to each variable, *J* is the Jacobian matrix, and *F* is a vector of partial derivatives of the corresponding variable with respect to the parameter of interest. The parameters we want to analyse are *k*_*S*_, *δ*_*D*_, *δ*_*R*_, *s*_*C*_, *k*_*E*_, *λ*_*E*_, *k*_*U*_, and *λ*_*U*_, as well as the initial conditions for each variable, *M*(0), *D*(0), *R*(0), *E*(0), *U*(0). The method is outlined in Atherton, Schainker & Ducot (1975). The relative local effect was measured by *S*_par,var_(*t*)∕var(*t*).

## Results

This section includes mathematical analysis of the system equilibria and the computational results of the model.

**Table 1:** Table of parameters.

Parameter	Value
*κ*_*S*_	2.7938
*δ*_*D*_	9.9626
*δ*_*R*_	0.0727
*κ*_*E*_	0.0961
*λ*_*E*_	0.0418
*k*_*U*_	5.0
*λ*_*U*_	0.3387
*s*_*C*_	9.517
*R*_0_	1/(1 + *ϵ*)
*ϵ*	10^−4^
*α*	1 + *ϵ*

**Table 2:** Data used in parameter estimation. Standard deviation is given after the mean as ±.

Cell viability, %			GAG, % non-impact		ATP, % Control average	
Time, h	7 J/cm^2^	14 J/cm^2^	Time, d	7 J/cm^2^	Time, h	7 J/cm^2^
0	100 ± 8	100 ± 8	7	81 ± 4	24	18 ± 18
1	80 ± 9	73 ± 4	14	87 ± 10	48	30 ± 19
2	74 ± 7	71 ± 8				
4	60 ± 13	60 ± 2				
6	65 ± 6	43 ± 2				
12	51 ± 7	42 ± 7				
24	52 ± 7	39 ± 4				
48	47 ± 7	39 ± 7				
72	52 ± 7	44 ± 7				

### Mathematical analysis

In deterministic mathematical models, like the present one, the equivalent of statistical analysis done for experimental data or for stochastic models is equilibrium analysis. We also analyze the local effect of parameter perturbations on the different variables through local sensitivity analysis.

The details of the equilibrium analysis are given in Appendix. Briefly, the non-trivial equilibrium is stable and will be determined by the effect of the oxidative stress on the cell viability. In other words, we expect to reach a new homeostasis with lower levels of cell viability and appropriate levels of ROS, ATP, and GAG. No chaos is present in the system.

### Numerical results and data fitting

**Figure 3:**
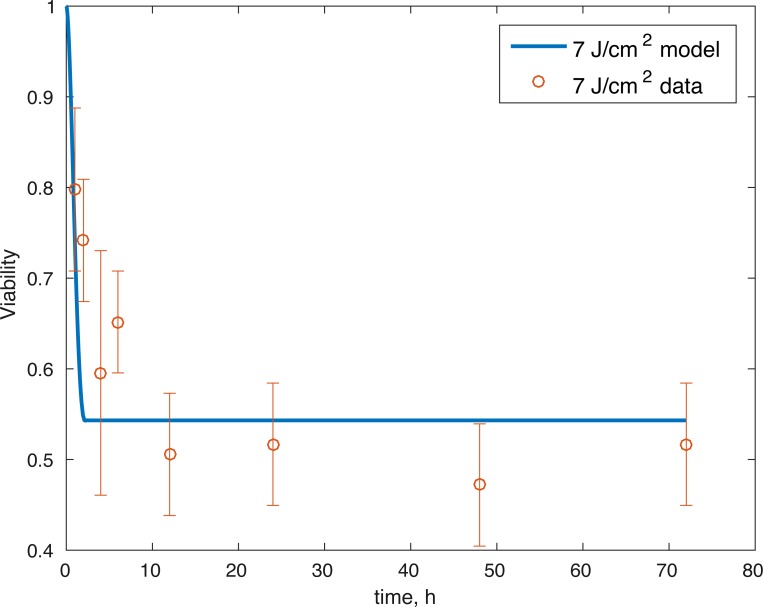
Relative proportion of live cells after an impact of 7 J/cm^2^ and its fit to available cell viability data from Martin et al. (2009) (open circles).

**Figure 4:**
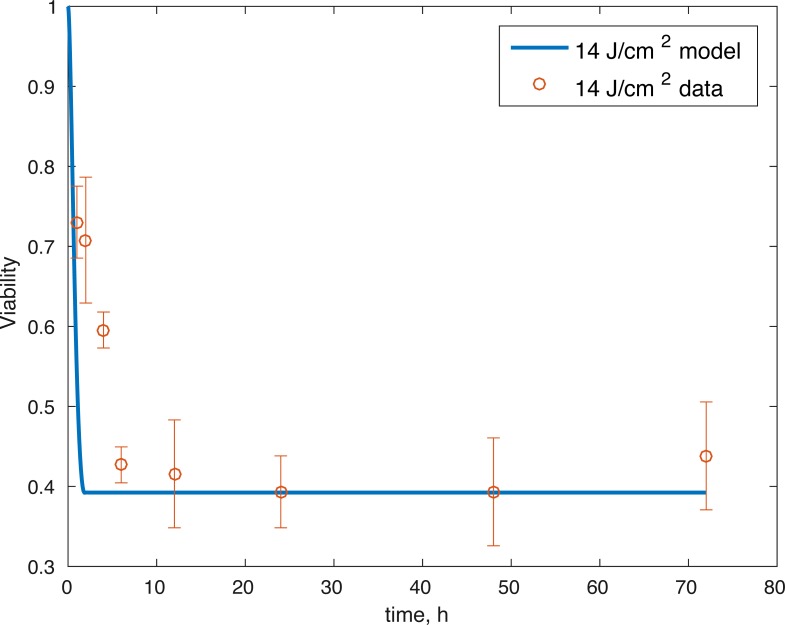
Relative proportion of live cells after an impact of 14 J/cm^2^ and its fit to available cell viability data from Martin et al. (2009) (open circles).

**Figure 5:**
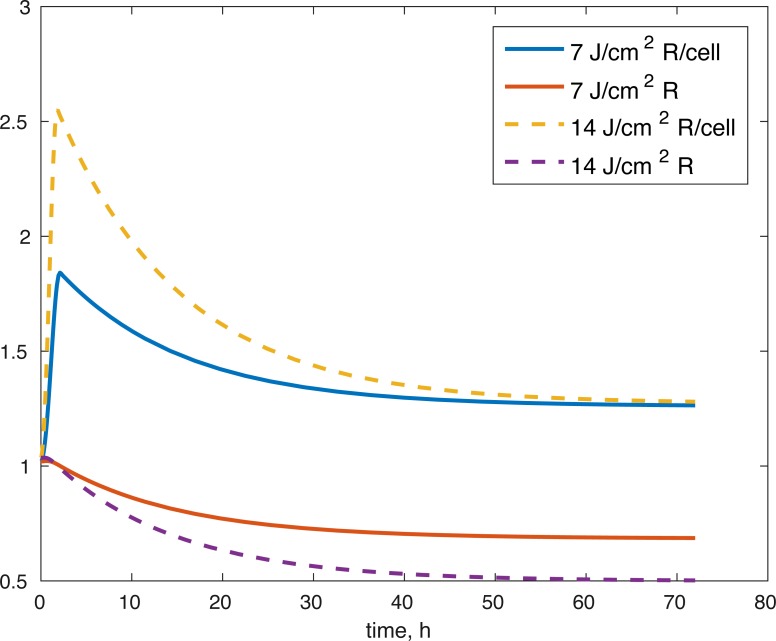
Projected relative concentration of available ROS after 7 J/cm^2^ impact, and 14 J/cm^2^ impact, after 72 h.

**Figure 6:**
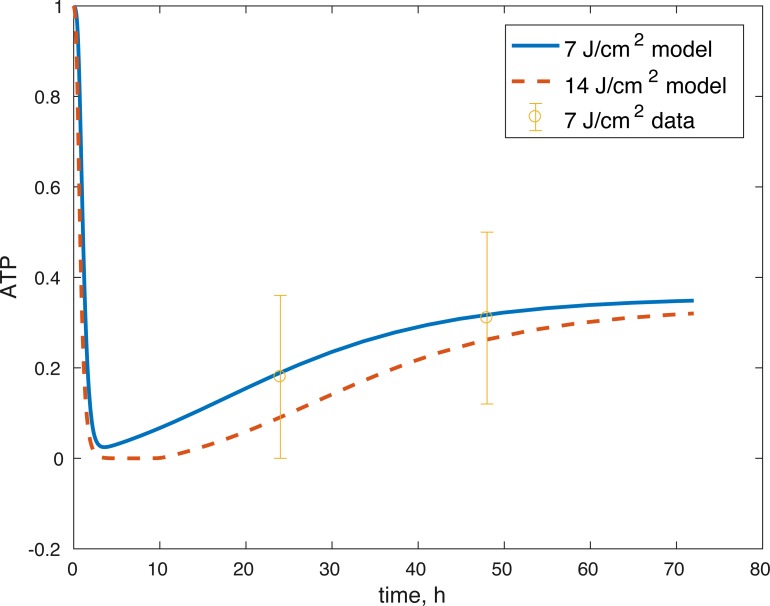
Projected relative concentration of available ATP after 7 J/cm^2^ impact and 14 J/cm^2^ impact, compared to 7 J/cm^2^ ATP data.

**Figure 7:**
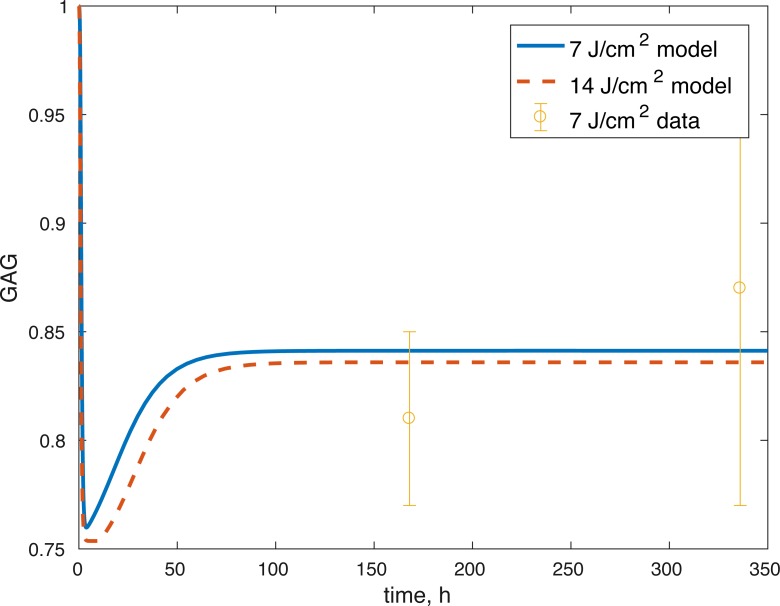
Projected relative concentration of GAG after 7 and 14 J/cm^2^ impact, compared to 7 J/cm^2^ data.

The initial conditions (cell viability and chemical concentrations at time = 0 h), in order (*M*(0), *D*(0), *R*(0), *E*(0), *U*(0)) for the 7 J/cm^2^ impact were (1, 0, 1.0202, 1, 1), and (1, 0, 1.036, 1, 1) for the 14 J/cm^2^ impact simulation. The root mean square error for the fit to the 7 J/cm^2^ data is 0.074, and to the 14 J/cm^2^ data is 0.123. The results are presented in Figs. 3–7. The total cell viability (functional plus dysfunctional mitochondria) fits well with the cellular viability presented in Martin et al. (2009), as evident from Figs. 3 and 4. The ATP simulation also fits well with the available data from Coleman, Buckwalter & Martin (2015) as seen in Fig. 6. The GAG simulation also fit well with the given data (Fig. 7). Overall, the model seems to capture the biochemical dynamics of the impact site of the cartilage explant.

### Sensitivity analysis results

**Figure 8:**
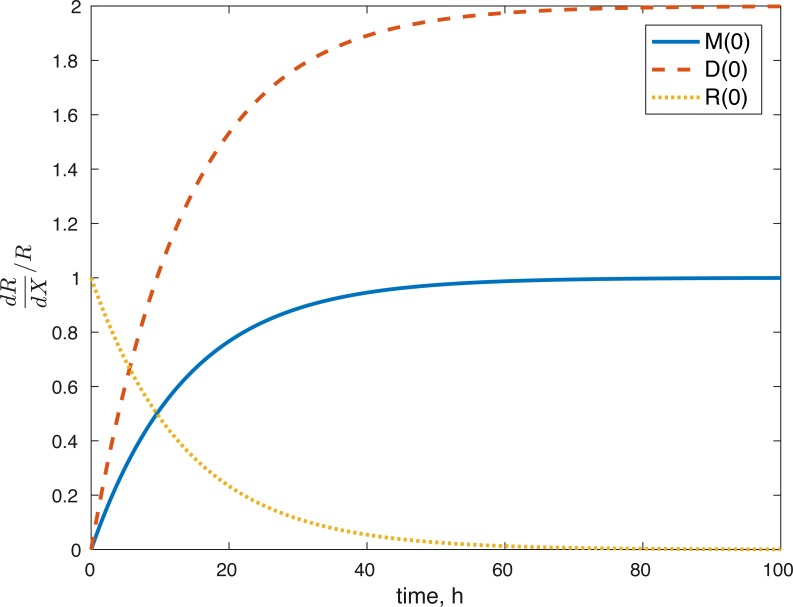
Relative sensitivity of ROS to initial conditions ( *X* = *M*(0), *D*(0), *R*(0)).

**Figure 9:**
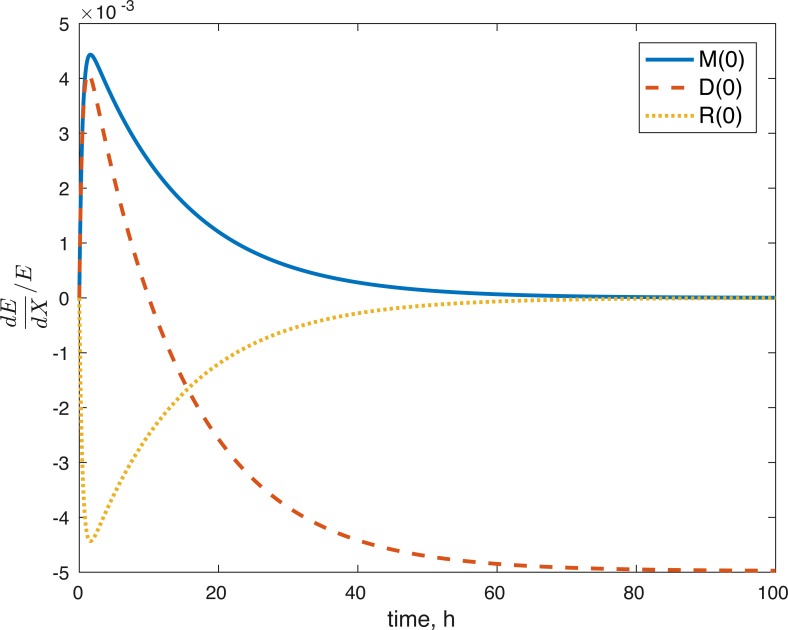
Relative sensitivity of ATP to initial conditions (*X* = *M*(0), *D*(0), *R*(0)).

None of the parameters affect any of the variables locally. The initial conditions had some effect, although none of them had a local effect on *U*. The values of *E*(0) and *U*(0) did not affect any of the variables. The effect of changes in *M*(0) on *M*(*t*) is constantly 1, which implies that the relative value of *M*(*t*) is aways dependent on *M*(0). The effect of *M*(0) on *D* is 0, and on *R* and *E* can be seen in Figs. 8 and 9. *D*(0) does not affect *M* and *D* and its effect on *R* and *E* can be seen in Figs. 8 and 9. Changes in *R*(0) affects *R* and *E*, as seen in Figs. 8 and 9, respectively.

## Discussion

We constructed a model of the effects of oxidative stress on the energy production and proteoglycan release of a cartilage explant after a blunt impact. The model considered the effect of the impact on the mitochondrial ROS release and the resulting disruption in ATP production, which in turn negatively affects GAG release and cartilage structure.

The model’s results fit well with the results of the laboratory experiments both qualitatively and quantitatively. The simulations seem to capture well the cell viability dynamics (Figs. 3 and 4), the amount of ATP available at the impact site post injury (Fig. 6), as well as the GAG content (Fig. 7). Particularly, they capture the recovery in ATP production after the initial cell death and disruption. They capture, to an extent, the GAG production recovery as well.

The fact that the model’s outcomes are not sensitive to the local perturbations of the variables implies that our system is stable, as suggested by the equilibrium analysis. Our system is sensitive to the initial conditions, which is understandable. The amount of ROS gradually becomes independent of the initial burst of ROS due to the impact, and dependent on the current viability of both cell types. This effect is seen in Fig. 8. At the same time, the amount of initial ROS has a significant effect on the amount of cells with dysfunctional mitochondria, *D*, which is not surprising given that we assume that oxidative stress leads to apoptosis. Significantly less of an effect was observed in the sensitivity of cells with functional mitochondria to the initial amount of ROS, so it was assumed to be 0. Overall, from the local sensitivity analysis, *R*(0) seems to have the largest effect on the system, which is understandable both from mathematical standpoint (we construct the model so that the energy of the impact is related to the amount of initial ROS) and biologically. There is evidence that mechanical strain increases ROS output in chondrocytes and that the relationship is almost linear (Brouillette et al., 2013). Therefore, measuring the impact strain can be predictive of the ROS release, which in turn can predict the loss of viability, metabolism (ATP) and structure (GAG).

Several limitations of our modeling efforts should be addressed. Scarcity of longitudinal data for ATP production and GAG availability means the model results support the underlying hypothesis about the effect of the post-impact oxidative stress on the biochemical functions in articular cartilage, but they do not allow us to conclude that the dynamics we describe are entirely accurate. Furthermore, the whole model is non-dimensional and the estimated parameters non-mechanistic, which makes the model only appropriate for estimating relative levels of the biochemical compounds as compared to control conditions. More data and measurements would be needed for addressing these issues. The parameters themselves were estimated and the error found may be a local minimum, rather than a global one, so other parameter sets might give us a similar fit. However, the idea that the impact changes the initial amount of ROS released in the tissue seems to work to validate the viability of cells after the 14 J/cm^2^ impact with a set of parameters estimated from the 7 J/cm^2^ impact. More data at that stronger impact level would be needed to validate our ATP and GAG predictions. Our predictions for the amount of ROS present (Fig. 5), both in the explant impact area, and per cell in the impact area, seem to qualitatively capture expectations, namely high amounts of initial ROS, which level off eventually, as seen in Goodwin et al. (2010). All variables within the model are measurable. Viability, ATP, and GAG are already measured and the ratio between cells with functional and dysfunctional mitochondria and ROS levels can be estimated using dihydroethidium, which stains for elevated ROS production, and confocal imaging and cell counting (Brouillette et al., 2013). Furthermore, the lack of local sensitivity of the model to its parameters suggests that once a measurable set of parameters is found, the variance in those parameters would not have a significant effect on the outcomes. The biological implications are that if we know a measurable set for the control outcome, then we could make predictions about the pathological outcome by looking at the impact’s mechanics (measured as energy or strain) and the resulting ROS levels. However, at this stage, this is future work.

A major result presented in Martin et al. (2009) suggests the positive effect of antioxidants, N-acetylcysteine (NAC) particularly, on the post-impact cellular viability and overall cartilage stability, as measured by GAG content. The fact that treating the cartilage explant with NAC results in mitigating the effects of the blunt impact, leads to the conclusion that reducing oxidative stress and mitochondrial dysfunction post-impact is a viable option for preventing the development of OA. Modeling the effect of NAC and the timing of its application will be the subject of further work. The current effort is focused on successfully establishing a set of equations that are capable of describing the control and the pathological outcomes.

Work on creating and implementing *in silico* models like the one presented here may have a significant role in predicting the harmful effect of impact on cartilage explants and eventually translate to predicting post-impact patient outcomes. A viable options is going from imaging the joint injury, which can predict the severity (mechanical stress) of the impact (Anderson et al., 2011). The physical strain is a predictor of the ROS release (Brouillette et al., 2013), which in turn, corroborated by the current model, is a predictor of metabolic function and cartilage stability and cell viability (Coleman et al., 2016; Goetz et al., 2017; Goodwin et al., 2010; Martin et al., 2009). This translation of models to patients will require merging of biomechanics and biochemistry: quantifying the relationship between cell death and chemical signaling due to external mechanical stress (Bartell et al., 2015), mapping the stress field for individual patients injury (Ateshian, Henak & Weiss, 2015), and creating a mathematical model that combines the two (Ayati et al., 2017; Kapitanov et al., 2016). Using mathematical models to describe and quantify the biochemical reactions that lead to cartilage damage after an impact, may eventually, once validation has been established, remove the need to run a large portion of repetitive laboratory experiments. An ODE system such as Eq. (1) is easily encapsulated in code (e.g., MATLAB) that can be used directly in the lab to predict experimental outcomes. The computations of the solutions to the system take on the order of seconds on contemporary desktop equipment, which can save significant experimental time and resources relative to conducting expensive, time-consuming, and error-prone experiments. However, in the near term more experiments are needed to inform models and for creating an accurate map of the important biochemical interactions.

##  Supplemental Information

10.7717/peerj.3468/supp-1 Supplemental Information 1 Matlab code

## Appendix: Equilibrium analysis

The $\vec{0}$ equilibrium exists. A general equilibrium solution is: any *M*^∗^ > 0, any *D*^∗^ > 0 (subject to the restrictions *S*(*R*^∗^) = 0 and the parameter relationships outlined in ‘Parameter Relationships’) and

\begin{eqnarray*}{R}^{\ast }= \frac{{\alpha }_{M}({M}^{\ast }+{k}_{D}{D}^{\ast })}{{\delta }_{R}} , \end{eqnarray*}

\begin{eqnarray*}{E}^{\ast }= \frac{{f}_{E}( \frac{{R}^{\ast }}{{M}^{\ast }+{D}^{\ast }+\epsilon } )}{{\delta }_{E}} , \end{eqnarray*}

\begin{eqnarray*}{U}^{\ast }=0~\text{or}~{U}^{\ast }= \frac{1+{\lambda }_{U}{E}^{\ast }}{1+{\lambda }_{U}} . \end{eqnarray*}

The *U*^∗^ = 0 equilibrium is unstable, so if we assume that *U*(0) > 0, it will not be reached. Therefore, we only need to focus on the positive *U*^∗^. As we have established, since *R*^∗^ ≤ 1, *S*(*R*^∗^) = *S*′(*R*^∗^) = 0. Therefore, the eigenvalues around the equilibrium are: *e*_1_ = *e*_2_ = 0, *e*_3_ =  − *δ*_*R*_, *e*_4_ =  − *δ*_*E*_, and

\begin{eqnarray*}{e}_{5}={k}_{U} \frac{1+{\lambda }_{U}{E}^{\ast }-2(1+{\lambda }_{U}){U}^{\ast }}{1+{\lambda }_{U}{E}^{\ast }} . \end{eqnarray*}

The *U*^∗^ = 0 equilibrium makes *e*_5_ positive, as expected from the logistic nature of the $ \frac{dU}{dt} $ equation in Eq. (1). Since we do not consider this equilibrium biologically viable in our case, let us consider the other equilibrium, ${U}^{\ast }= \frac{1+{\lambda }_{U}{E}^{\ast }}{1+{\lambda }_{U}} $. The eigenvalue *e*_5_ =  − *k*_*U*_ in this case, so is also negative. Therefore, the equilibrium is non-hyperbolic with *e*_1_ = *e*_2_ = 0 and *e*_3_, *e*_4_, and *e*_5_ negative.

A simple linearization is sufficient to establish that the variables *M* and *D* form the center subspace of the system, while *E*, *U*, and *R* form the stable subspace of the system. Because of the form of *S*(*R*), the equilibrium of the system is not approached in the usual manner when considering analyses of systems of equations. If *R* = 1, then $ \frac{dM}{dt} $ and $ \frac{dD}{dt} $ are 0, which establishes *M*^∗^ and *D*^∗^. Then *R*^∗^ is determined by *M*^∗^ and *D*^∗^. Therefore, in order to determine the flow of the central subspace, we need to examine its behavior as *R* → 1^+^. For any *R* > 1, the system determined by the central subspace and the assumption on *R* is:

\begin{eqnarray*} \frac{dM}{dt} =-{k}_{S}M{s}_{C}(R-1)^{\alpha } \end{eqnarray*}

\begin{eqnarray*} \frac{dD}{dt} ={k}_{S}M{s}_{C}(R-1)^{\alpha }-{\delta }_{D}D{s}_{C}(R-1)^{\alpha } \end{eqnarray*}

A simple change of variables *M* = *M* − *M*^∗^, *D* = *D* − *D*^∗^ results in

(4) \begin{eqnarray*} \frac{dM}{dt} =-{k}_{S}(M+{M}^{\ast }){s}_{C}(R-1)^{\alpha }\end{eqnarray*}

(5) \begin{eqnarray*} \frac{dD}{dt} ={k}_{S}(M+{M}^{\ast }){s}_{C}(R-1)^{\alpha }-{\delta }_{D}(D+{D}^{\ast }){s}_{C}(R-1)^{\alpha }\end{eqnarray*}

for which $\vec{0}$ is the equilibrium (keep in mind *R* → 1^+^ is fixed). The eigenvalues of this system are −*k*_*S*_*s*_*C*_(*R* − 1)^*α*^ and −*δ*_*D*_*s*_*C*_(*R* − 1)^*α*^, both negative. Therefore, the equilibrium (*M*^∗^, *D*^∗^) is asymptotically stable, which implies that the equilibrium of the original system is also asymptotically stable.

## Additional Information and Declarations

### Data Availability

The following information was supplied regarding data availability: The raw data used for simulations is included in the manuscript in Table 2 and the Matlab code is available as Supplemental Information 1.
